# Positive emotions foster spontaneous synchronisation in a group movement improvisation task

**DOI:** 10.3389/fnhum.2022.944241

**Published:** 2022-08-30

**Authors:** Andrii Smykovskyi, Marta M. N. Bieńkiewicz, Simon Pla, Stefan Janaqi, Benoît G. Bardy

**Affiliations:** EuroMov Digital Health in Motion, University of Montpellier, IMT Mines Ales, Montpellier, France

**Keywords:** interpersonal synchronisation, joint action, emotion, improvisation, non-verbal behaviour

## Abstract

Emotions are a natural vector for acting together with others and are witnessed in human behaviour, perception and body functions. For this reason, studies of human-to-human interaction, such as multi-person motor synchronisation, are a perfect setting to disentangle the linkage of emotion with socio-motor interaction. And yet, the majority of joint action studies aiming at understanding the impact of emotions on multi-person performance resort to enacted emotions, the ones that are emulated based on the previous experience of such emotions, and almost exclusively focus on dyadic interaction. In addition, tasks chosen to study emotion in joint action are frequently characterised by a reduced number of physical dimensions to gain experimental control and subsequent facilitation in data analysis. Therefore, it is not clear how naturalistically induced emotions diffuse in more ecological interactions with other people and how emotions affect the process of interpersonal synchronisation. Here, we show that positive and negative emotions differently alter spontaneous human synchronous behaviour during a multi-person improvisation task. The study involved 39 participants organised in triads who self-reported liking improvisational activities (e.g., dancing). The task involved producing improvisational movements with the right hand. Participants were emotionally induced by manipulated social feedback involving a personal ranking score. Three-dimensional spatio-temporal data and cardiac activity were extracted and transformed into oscillatory signals (phases) to compute behavioural and physiological synchrony. Our results demonstrate that individuals induced with positive emotions, as opposed to negative emotions or a neutral state, maintained behavioural synchrony with other group members for a longer period of time. These findings contribute to the emerging shift of neuroscience of emotion and affective sciences towards the environment  of social significance where emotions appear the most—in interaction with others. Our study showcases a method of quantification of synchrony in an improvisational and interactive task based on a well-established Kuramoto model.

## Introduction

Rhythm entrainment is a social glue that helps humans to function and cooperate in large groups, constituting societies (Duranton and Gaunet, 2016). Since ancient times, people have engaged in rhythmic activities, such as singing or dancing, to improve their collective performances, such as hunting (von Zimmermann and Richardson, 2016). More recent historical records disclose that the introduction of synchronous drills into the Dutch Armed Forces in the late 16th century led to building a higher spirit of camaraderie and resulted in developing effective military units on the battlefield that altogether provided an edge for the Dutch army over other armies of the continent (McNeill, 1995). Interestingly, the drilling that made units more effective was believed to have established an emotional resonance between individuals. Consider this testimony of a person engaged in such drills: “Words are inadequate to describe the emotion aroused by the prolonged movement in unison that drilling involved. A sense of pervasive well-being is what I recall; more specifically, a strange sense of personal enlargement; a sort of swelling out, becoming bigger than life, thanks to participation in a collective ritual.” (McNeill, 1995, p. 2). This quote draws attention to a surprising interplay between synchronous movements and human emotions, which is the object of the present study.

Synchronisation examples are everywhere around and inside us: from planets orbiting in different galaxies to fireflies firing in perfect unison to pacemaker cells in the human heart. The emergence of spontaneous order is a result achieved through order in space and time. While spatial ordering can be explained and demonstrated by the development of tangible and frequently understood architectures, temporal ordering is more elusive (Strogatz, 2003). For synchronisation to occur, several conditions must be met, such as the production of individual rhythm or oscillations, behavioural proximity, allowing for the exertion of physical or chemical influence and the coupling function (Strogatz, 2003). Fireflies communicate with flashing lights, have their inner rhythm, and adjust it in response to the flashing of others to attain synchronisation. In this light synchronisation analysis is based upon the extraction of individual frequencies and their relation to the group frequency, as well as the extraction of phases, i.e., moments where an event occurs in a time series (Pikovsky et al., 2001).

The phenomenon of human emotion intrigued ancient philosophers and has progressively transformed into a modern field of psychological research. However, the field is suffering from ongoing challenges. The realisation that “There will never be an integrative theory of emotion…” (Niedenthal and Brauer, 2012, p. 275) is a surprising way to start the conclusions section of recent Annals publication on the social functionality of human emotion. The difficulty is that there are myriad ways to approach the concept of emotions depending on research interests such as relevance for survival, the implication of cognition, morality, social engagement, or even the tendency for action that gives or seizes power. Despite some agreements on such essential topics as whether to approach emotion as discrete, separate entities (e.g., anger, surprise, and happiness) or in terms of dimensions (e.g., valence, arousal) as well as agreement on the distinction of emotion from other affective states (Ekman, 2016), there is no widely accepted definition after already 100 years of research (Russell, 2012). This poses a problem because the progress in studying a concept with fuzzy borders and subsequent experimental studies can be ineffective without an accurate and clear definition. Thus, it is of utmost importance when studying emotions to outline the aimed concept to explain. We phenomenologically understand emotions as relevance detectors occurring at behavioural, cognitive and physiological modalities that guide the decision process (Sander et al., 2003). Emotion interpretation as a relevance detector has essential consequences concerning the emotion induction method, which will be discussed in more detail in the relevant section. There is, however, an agreement (Sander, 2013) that emotions are a complex phenomenon resulting from clearly identified stimuli and followed by a reaction that brings about change not only in the cognitive modality but is expressed through other modalities as well, such as behaviour and physiology, of relatively short duration. This exemplifies the need to consider the different modalities of human experience for understanding emotions.

Previous research shows that there is a higher chance of losing rapport and creating misunderstandings in the absence of synchronous rhythmical movements. The rhythmic convergence occurring spontaneously between people, without conscious and deliberate efforts to develop synchrony, was reported in experimental studies in which individuals, while sitting next to each other, tend to synchronise swings of their legs (Schmidt et al., 1990), rocking in chairs (Richardson et al., 2007), and side-by-side walking (Nessler and Gilliland, 2009). Interestingly, interpersonal motor synchronisation was found to increase social affiliation (Hove and Risen, 2009), enhance self-esteem (Lumsden et al., 2014), and develop the ability to cooperate (Valdesolo et al., 2010) and even improve tolerance to pain (Tarr et al., 2015). Once we understand the benefits of perceiving movement synchronisation, a logical question is how humans perceive synchronisation? In a dyadic Tai Chi experiment, it was found that the perception of phase synchrony, among other metrics such as mutual information or dynamical time warping, is the most prevailing metric humans use to identify synchronisation (Bente and Novotny, 2020). Continuing this line of thought, one might ask what comes first, is it the affect that causes improved synchronisation or is it the synchronisation that brings about a pleasant feeling? Fujiwara and Daibo (2018) found no evidence that changes in valence produced tangible changes in synchronisation levels. The authors supposed that one possible reason for the absence of effect is that emotion induction did not produce affective changes. Indeed, it is possible that watching parts of movies or specific picture sets, while being an effective induction procedure for some individuals, is not personally relevant when standardised for a multitude of different people. Even if the change in valence occurs, it does not automatically imply that emotion was evoked. Emotion does not only portray changes in valence (Quigley et al., 2014), which alone would describe mere changes in preferences (Scherer, 2005), but they activate special machinery acting through different modalities to prepare the body for the execution of specific actions (e.g., anger—to attack, fear—to run away, and sadness—to seek for help), the process referred to as action tendencies (Frijda, 2007).

The link between synchronisation and physiological arousal was empirically tested previously. For instance, watching a family member engaged in a dangerous activity, the heartbeat of both people tends to synchronise (Konvalinka et al., 2011). Similarly, during dialogue, the brain waves of the listeners tend to synchronise with the speaker’s (Hasson and Frith, 2016). What is not yet clear is how the emotional state impacts motor synchronisation. Besides a handful of studies (e.g., Varni et al., 2010; Paxton and Dale, 2013; Tschacher et al., 2014; Fujiwara and Daibo, 2018), the link between emotion and synchronisation remains largely unexplored, and the results are inconsistent. For instance, while Fujiwara and Daibo (2018) did not find evidence for an influence of affective valence on synchrony in a dyadic interaction, Varni et al. (2010) documented that the positive emotion (i.e., pleasure) facilitated synchronisation within a group of musicians in comparison to the negative emotion (i.e., anger).

In a recent review (Bieńkiewicz et al., 2021), we emphasised the need for reconciliation between emotion and joint action research. In line with this proposal, this study aims to show the effects of experimentally induced emotional valence (positive emotion vs. neutral state vs. negative emotion) on group motor synchronisation (arm movements between three people). Given the exploratory nature of this work, we held no quantified expectation about the amount of interactional change following emotional induction but a general hypothesis about the direction of this relationship. Based on the state-of-the-art reviewed above, we expected that positive emotions would improve synchrony and that negative emotions would disrupt it.

### Considerations for emotional induction in the lab

In this study, we used a method for emotional induction based on manipulated social feedback. Fundamentally, regardless of the chosen emotion induction method, it is a trade-off between ecological validity and experimental control (Levenson, 2003). Conservative procedures, such as watching video clips (Fakhrhosseini and Myounghoon, 2017), displaying powerful or amusing photos or listening to music, do not entail personal implications. For this reason, we assessed that those methods are suboptimal for our experiment, which concerns the manifestations of emotion in movement and cardiac signals. There are other methods to induce emotions such as autobiographic recall, also known as relived emotions, imagery of specific events and situational procedures such as adapting certain facial expressions to evoke a particular emotion, changing the environmental conditions such as heating the room to an uncomfortable temperature to induce anger, or to provide an enjoyable gift to induce happiness (Siedlecka and Denson, 2019). Deception studies, such as Harmon-Jones and Sigelman’s (2001) work on anger and aggression, arguably provide the closest to a real-life type of emotional induction in laboratory settings. The approach we have used in our study that affords high ecological validity relies on deception, based on success-failure manipulations owing to the personal involvement of participants in the process (Nummenmaa and Niemi, 2004), coupled with a picture type of emotion induction.

In this approach, each participant’s performance is subjectively evaluated by other members and followed by the presentation of a manipulated (in predetermined order) feedback of being perceived as the best, a tie, or the worst. The feedback is directed explicitly at the individual and their efforts, making the induction more personal. On top of that, if the task based on which the person receives feedback is of personal value, then the induction is expected to generate even a greater effect.

In summary, the original contribution of the present research is an experimental paradigm for studying the natural interplay between synchrony and emotion (positive, neutral, and negative), in the different modalities of behavioural, psychological, and physiological modalities, based on a new analytical tool for adjusting raw, three-dimensional and non-stationary signals to extract phase synchronisation. Specifically, we used a social feedback emotion induction technique based on a personally relevant task. Moreover, we developed an experimental paradigm in which participants explored space in three dimensions (instead of one or two) and suggested a five-step process to analyse group synchronisation for *N* > 2.

## Materials and methods

### Participants

Thirty-nine adults (21 females, mean age = 25.4 years, SD = 5.7) participated in the study. Participants were volunteers (authors’ network and students from various universities in Montpellier) who self-reported enjoying dancing as their leisure pastime or were professional dancers. Thirty-six were right-handed, and three were ambidextrous and obtained an Edinburgh Handedness Questionnaire (EHQ) score >–50. All participants were naïve to the goal of the study. They reported having no physical disabilities of any kind and had normal or corrected-to-normal vision. They did not benefit from any financial or material indemnity and participated with the goal of the advancement of scientific knowledge.

Participants were organised into groups of three people (triads) to perform the task together. These triads were randomly generated in groups of same-sex participants with no prior acquaintances within either of the group members. Most participants (*N* = 33) had no prior improvisational (e.g., dancing, gymnastics) experience other than leisure pastime, and six participants had intermediate experience with occasional group performances.

Before the start of the experiment, all participants read the information letter and gave written informed consent. The Institutional Review Board (IRB #2002A) approved the research protocol of the EuroMov Digital Health in Motion research unit, Montpellier, France.

### Experimental setup

The experiment was held in the Motion Capture laboratory of EuroMov. Calibration and data acquisition were performed with the closed shutters daily before the experimental sessions. Body kinematics were recorded with the Vicon motion capture system (Nexus MX13 Vicon System), with eight infrared cameras and a sampling frequency set at 100 Hz. In the experiment, we used nine retroreflective markers placed on each participant, but in the light of the research question addressed in the present article, we used only three markers: right forehead (RFHD), left forehead (LFHD), and right head of the second metacarpal bone (RFIN), providing global information about hand movement. Those body points were selected during pilot runs as meaningful for extracting the participants’ body kinematics for the computation of group synchronisation. In addition, we captured participants’ cardiac activity *via* the Delsys Trigno wireless biofeedback system with Avanti EKG sensors (placed according to Delsys guidelines). The sampling frequency of cardiac data acquisition was set to 2,148 Hz, with recordings being triggered by the analogue board of the Vicon System to synchronise temporal data alignment. We used the Delsys Trigno Research + system for synchronous recordings of multiple devices. The Vicon system was connected to the Delsys system *via* the Vicon analogue board and a Delsys trigger box. The Delsys System was launched once from the beginning until the end of the experiment. Each time a Vicon recording was launched, the Delsys trigger generated an electrical spike to start acquiring ECG data and dropped to baseline level when the recording stopped. Afterward, these spikes in activity were used to identify the start and end of the trial. The Delsys ECG electrode signal and the Vicon motion capture data were automatically aligned regarding time stamps *via* the Vicon analogue board. Mobile devices were not connected to the Delsys as they were controlled from the Mentimeter website^1^ and launched manually for all the participants at the exact moment. We developed a Python code to change the slides simultaneously. For this, we needed to keep the different Mentimeter presentations for each of the participants on separate tabs within a browser. With each click of the letter R, a series of commands were generated: Ctrl 1 (to open the first tab), then right arrow (to go to the next slide), then Ctrl 2, then right arrow, then Ctrl 3 and right arrow.

Before each experiment, the participant number was attributed to each individual in order to anonymise their data for processing. Participants were invited to stand in the part of the triangle in all trials, on a demarcated space for each participant (1.2 m distance in a straight line from each other), with the right hand slightly extended toward the centre of the formed equilateral triangle (angles 60° each). Participants were also asked to charge and bring their phones to the experiment to be used for the emotional induction procedure.

### Experimental task

**FIGURE 1:**
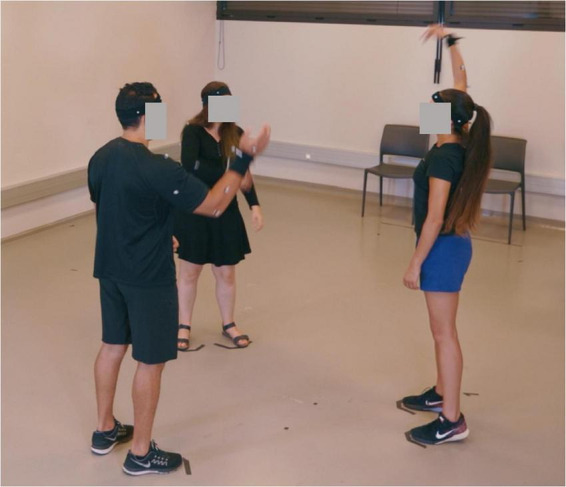
Experimental setup with three participants engaged in the movement improvisation task.

The current experiment was developed based on the mirror game paradigm for dyadic interaction (Noy et al., 2011) and extended to a group interaction (Himberg et al., 2018). Importantly, in our experiment (Figure 1), no specific instruction to synchronise was provided. This was to attribute the rise in synchronisation level to spontaneous entrainment between participants. However, during the trial, we kept the original, explicit instruction used in the modified mirror game task to create complex, varied and interesting movements with the right hand. Additionally, we instructed participants not to communicate during the experiment. Finally, participants were asked not to move their feet and only to express themselves with the movements of their right hand.

### Experimental procedure

Before coming to the laboratory, the participants received general information about the experiment. To them, the experiment was about personality extraction from movement and the development of group synergies in dancing. Upon arrival at the laboratory and after giving their written informed consent, they were equipped with the motion capture markers and instructed on how to place the Delsys Trigno ECG sensor correctly. Next, the accuracy of the recorded ECG data and specifically a salient QRS complex was verified visually before the experiment’s launch. There were 15 trials of 30 s each (7 min and 30 s in total). The order of trials was randomised and counterbalanced across groups. Specifically, two random orders of trials were generated; seven triads were induced accordingly to the first order and six triads were induced accordingly to the second order. Jumping or turning around during the trials was not allowed.

After each trial, participants were asked to rank the performance of each triad member (including themselves) for that particular trial. When all scores were submitted, each participant would receive a (pre-programmed) feedback score for their own performance coming from their triad mates. This feedback based on the group evaluation of one’s performance was part of the emotion induction method. The detailed emotion induction method, cover story, and rationale are described in section “Emotion elicitation”. Following each trial, all three participants received the same feedback (i.e., all positive, neutral, or negative). Participants could not see the others’ scores and thus were unaware that the (pre-programmed) results were the same for everyone.

After each emotion induction, we sought to detect resulted changes in the following trial. A particular case was the first trial which was not included in the analysis because there was no emotion induction before it. As of the second trial, the participants began to evaluate each other’s performance in the preceding trial and received an emotion induction followed by a trial that uncovered the induction effect.

**FIGURE 2:**
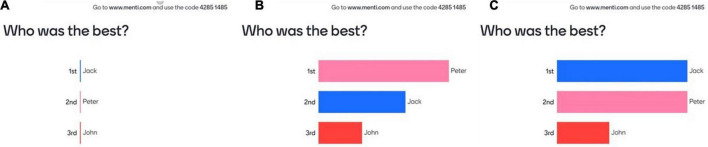
The first screen was visible before the experiment started to explain to the participants how the feedback worked, first before the votes **(A)**, then after the polls **(B)**, with the possibility that sometimes the scores could be tied **(C)**.

The ranking was performed using the participants’ own phones connected to menti.com. Mentimeter is a website designed for interactive presentations but was precisely adjusted here by the experimenter for emotion elicitation. Once connected to the Mentimeter presentation, the computer screen was shown to illustrate the outcome of the triad vote (Figure 2). Before starting the experiment, participants were asked to answer the questions *via* menti.com and familiarise themselves with the process of answering them on their smartphones.

**FIGURE 3:**
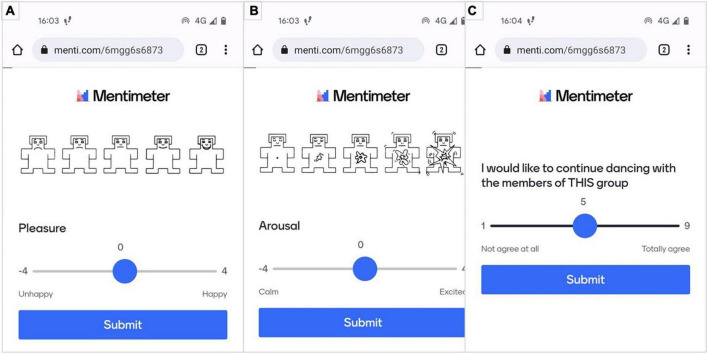
Evaluation of emotional [**(A)** pleasure; **(B)** arousal] and social **(C)** consequences after each performed trial.

When feedback was submitted, three more questions were prompted on a screen to evaluate two emotion scales—pleasure and arousal—and their social consequences (see Figure 3). The first two questions were the scales of affective dimensions derived from the Self-Assessment Manikin (Bradley and Lang, 1994) on valence (Figure 3A) and arousal (Figure 3B) for assessment of the emotion associated with the participants’ performance and the manipulated feedback they received. The third question (Figure 3C) assessed the social consequences of the preceding emotional elicitation. In part, it contains the motivational aspect of the individual to perform the task. For this article, the participants’ Mentimeter is presented in English, whilst participants received their feedback in French.

Then, the experimenter discussed with participants their impression of the experiment to verify whether anyone understood that obtained feedback was manipulated. Finally, the deception procedure for emotion elicitation of the study was uncovered for participants, as well as the necessity of such a deception design.

### Emotion elicitation

All participants were subjected to a social deception procedure with a cover story presented before the launch of the experiment that all participants in the triad would be asked to provide rankings of the performance of their triad mates and receive one themselves, based on the esthetical value and complexity of their movements. Being ranked “the best” provides three points, “second” two points and “third” one point. In such a manner, the range of possible points was between three (everyone ranked a person as the worst) and nine (everyone ranked a person as the best). The cumulative score of rankings of three participants was said to be automatically calculated and then provided personally to each individual.

**FIGURE 4:**
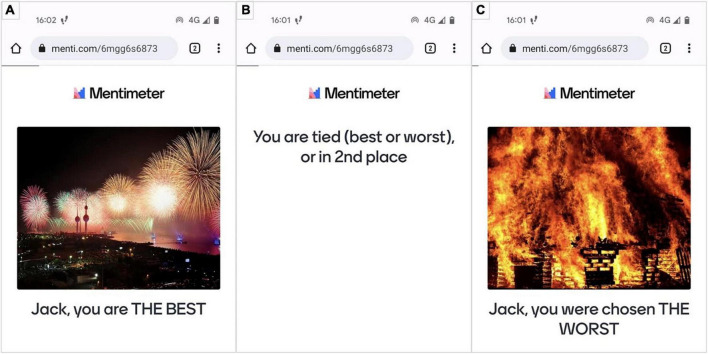
Emotion induction with positive feedback and positively valenced image from the OASIS database **(A)**, neutral state **(B)**, and negative emotion induction with negatively valenced image from the OASIS database **(C)**.

As stated in the introduction, the emotion induction method is a compromise between ecological validity and experimental control, and we discuss the two aspects. Firstly, for the induction method to have high ecological validity, the goal is to create an elicitation environment in which the induction is personally relevant. For this reason, the task in which the participants are engaged must be of importance to them. Our experimental task is based on expressive improvisation dancing. The emotional induction was achieved through feedback concerning perceived dancing performance. The feedback starts with the participant’s name to render the evaluation person-oriented. Specifically, after each trial, the participants received personalised positive or negative feedback: “Name, you are the BEST” (Figure 4A) and “Name, you were chosen the WORST” (Figure 4C). Receiving evaluative feedback from competent peers of being chosen as “the best” in a personally valuable task (i.e., dancing for dancers) would result in a more positive affect. Conversely, being selected as “the worst” would result in a more negative affect. Moreover, to enhance the feedback’s evaluative form and facilitate interpretation by making the evaluation more salient, the words “BEST” and “WORST” were in capital letters. In addition, to reinforce the induction, we used images from the Open Affective Standardized Image Set (OASIS) (Kurdi et al., 2017) of the same arousal level but opposite valence level. In this way, the positive visual stimulus (e.g., fireworks) has a valence score of 6.27 and arousal of 4.98; the negative stimulus, e.g., parts of a house on fire, has a valence score of 1.73 and arousal of 5.28.

The control condition was used as a baseline. We opted for feedback that contained uncertainty for the participants: They would receive the feedback “You are tied (best or worst) or in 2nd place” (Figure 4B). We expected that this ambiguity of possibly being the best and the worst simultaneously would result in a more neutral induction.

### Variables, data processing, and statistics

For our independent variable, there were three experimental conditions for eliciting emotions (within-subject factor): positive emotion (P), neutral state (Nt), and negative emotion (Ng). The stimulus was the feedback received on the smartphones about the ranking score. This score was manipulated, and everyone in the triad received *de facto* the same feedback.

For our dependent variables, three modalities were analysed: behavioural [i.e., Time-in-Synchrony (TIS) scores of velocities], psychological (i.e., Self-Assessment Manikin (SAM) scale (Pleasure, Arousal) and Social Consequences scores), and physiological (i.e., TIS of time series of intervals between two successive heartbeats called the RR interval).

#### Behavioural data

The three-dimensional coordinates of hand movements were acquired. The hand was decided to be used as a reference point because the task consisted of hand movements, and the information provided by the cinematics of the hand marker was enough to extract the movement richness in the improvisational task. Then, the data were inspected and labelled. Data inspection was performed with the software Vicon Nexus 1.8.5. The labelling of each of three retro-reflective markers (one on hand and two on the head) was performed manually. If the gaps in trajectories were identified, the built-in gap-filling spline function was used.

Several mid-layer variables were calculated from these raw data: coordinate velocities, accelerations, and angles of head markers, which were later used to compute our metrics.

##### Velocity calculation

The total velocity is the norm of the coordinate velocity vector that is if we note by *P*(*t*) = [*x*(*t*),*y*(*t*),*z*(*t*)] the position of a marker at the time *t*, then the coordinate velocities are

$\left[\frac{d\,x}{d\,t},\frac{d\,y}{d\,t},\frac{d\,z}{d\,t}\right]$ and total velocity

$v\,\left(t\right)=\sqrt{{\left(\frac{d\,x}{d\,t}\right)}^{2}+{\left(\frac{d\,y}{d\,t}\right)}^{2}+{\left(\frac{d\,z}{d\,t}\right)}^{2}}$

The issue with raw velocities is that the anthropometric data influence their calculation. Therefore, we normalised the data before metric calculation. In this experiment, the participants formed a triangle. Each triangle angle was indicated with a special spot on the ground of the laboratory, indicating a specific place for participant one, two and three. Each participant was always occupying the same physical place in all trials. We used three reflective markers placed on the floor, one for each participant, before the start of data acquisition. By keeping the setup consistent across different triads, we were able to use the position of three markers placed on the ground as reference points for the local coordinates of three participants. Then, for each participant, we shifted and rotated each of them to a particular point in space. Then their limb movements were normalised. The maximal distance between the head and hand marker was calculated for each person. Then all position data were multiplied in order to bring this maximal distance to the same value for all three participants. This allowed neglecting the anthropometric differences between individuals.

**FIGURE 5:**
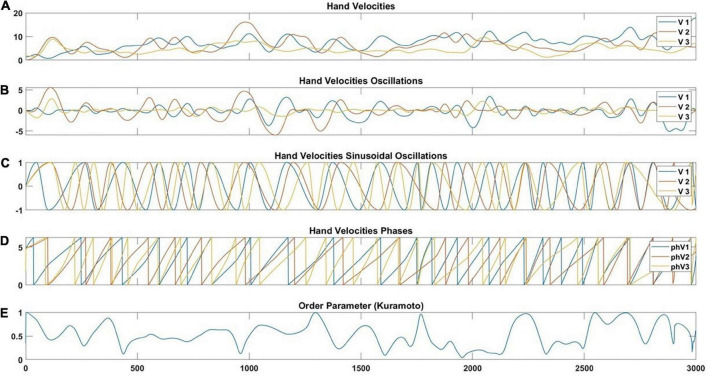
Illustration of the velocity pre-processing steps. Taking the raw signal **(A)**, implementing local detrending **(B)**, and the undulatory part of the signal **(C)** are used to extract phases with the Hilbert Transform **(D)** and implementation of the Kuramoto order parameter **(E)**. Within the graph legend, the V1, V2, and V3 correspond to the velocity of participant 1 (V1), participant 2 (V2), and participant 3 (V3). In addition, the phV1, phV2, and phV3 correspond to the we extracted phase velocities of participant 1 (phV1), participant 2 (phV2), and participant 3 (phV3).

Firstly, the original velocities (Figure 5A) were extracted for each trial. Secondly, local detrending was performed to detect the oscillations by bringing the inflexion points to 0. When we compare the velocity variation between individuals, it is not the amplitude of that variation that counts but the oscillatory part. In simpler terms, a large movement of a particular oscillation would be similar to a small movement of the same oscillations. In order to extract the oscillatory part of the movement, we were inspired by the detrending techniques that we adapted to local levels. Schematically, for a given signal s⁢(t) we first calculate the inflection points (*t*(*k*),*s*(*t*(*k*)) that is such as *s*″(*t*(*k*)) =  0. In other words, we detect instances where a second derivative of the position time series equals 0. Then, we create a piecewise linear curve *L*(*t*) by joining successive (*t*(*k*),*s*(*t*(*k*)) by linear interpolation. This permits calculating the detrended signal *S*(*t*) = *s*(*t*)−*L*(*t*). This operation brings the inflexion points to 0 (Figure 5B).

The next step was to bring the local maxima of *S*(*t*) to *1* and the local minima of *S*(*t*) to –1. These successive new maxima and minima were connected by a local sine-like function (Figure 5C). The phases of these oscillatory signals were extracted with the Hilbert transform (Figure 5D). Finally, we calculated the Order Parameter of Kuramoto (Figure 5E), which quantifies the proximity of phases. Let θ_1_(*t*),θ_2_(*t*),θ_3_(*t*) be the phases of 3 individuals at time *t*. The vectors *p*_*k*_(*t*) = *exp*(*i*×θ_*k*_(*t*)),*k* = 1, 2, 3 have modulus 1. The Order Parameter at time *t* is the modulus of the mean vector.


$$\mathrm{opmt}\,\left(t\right)=\left|\frac{1}{3}\,\sum_{k=1}^{3}p_{k}\,\left(t\right)\right|$$

Our metrics of interest—time in synchrony (TIS)—were computed from the phase data. A significant duration threshold of 3 s (10% of trial duration) was used to consider that TIS were valid. Another important point is the thresholds of TIS values. We calculated the probability distribution of Order Parameter values for a given triad through all its trials. Then, the TIS of order parameter was extracted according to the IQR principle, where Q1 represented the lower threshold for the weak synchronisation (less than or equal to 75% of pdf data), Q2—medium synchronisation (50% of data), and Q3— strong synchronisation (25% of data). In this study, we analysed the three levels of synchronisation, and prioritising, however, the results obtained with the weak level. The first reason is based upon the exploratory nature of using the Kuramoto model for the identification of affective information. The second reason relates to the non-linear dynamics literature on human synchronisation, in which humans are often regarded as weakly coupled oscillators (Pikovsky et al., 2001) as opposed to mediumly coupled (e.g., humans with haptic contact such as walking hand in hand) or strongly coupled oscillators (e.g., car wheels connected by the car axle).

In order to verify whether the effect found was not due to chance, we randomly generated new fake triads, or pseudogroups, and compared them with the experimentally obtained data. Pseudo groups were organised by shuffling velocity time series from all triads and all trials and then randomly pairing them. For instance, one pseudo trial could be organised by pairing velocity time series from Participant (1) Triad (1) Trial (1) with Participant (3) Triad (11) Trial (9) and Participant (3) Triad (4) Trial (15). Afterwards, the other velocity time series were shuffled and paired until all the pseudo trials were generated.

Finally, to control that the task was executed correctly and that the participants were paying attention to each other, we dynamically built a triangle out of the head data for each individual. First, the centre point between each pair of LFHD and RFHD was found. The left-head to right-head points define a parallel to face vector *f*. Then a perpendicular vector to *f* was derived to detect the facing direction (head orientation). For each pair of participants (*i*,*j*) there were two numbers extracted. Pair *i-j* means participant *i* is facing participant *j* and pair *j-i* means that participant *j* is facing participant *i*. There were six pairs of numbers, two for each participant (e.g., for participant one: pair 1-2 and pair 1-3). The angles were calculated for each participant’s directedness into the triangle. For the clarity of calculation, the angles were transformed into percentages. For example, the value of 0.5 means that the head orientation was exactly in the centre of the triangle. For a dynamical triangle for this specific angle with an angle of 80°, the 0.5 value means that the facing angle is 40°. The closer the value is to 0 for one pair, the more this first participant orients their head toward the second person. If the value is 1 or above, it means that the person is looking at the opposite person or even further outside the triangle. Negative values are not possible. We arbitrarily chose the value of 0.6 as a threshold for head orientation inside the dynamical triangle.

#### Psychological data

To detect how successful was the induction, we used Self-Assessment Manikin (SAM) scale (Bradley and Lang, 1994). It is a graphical and non-verbal procedure that portrays human-like figures with different faces and sizes for two different scales: pleasure and arousal. For the valance scale, figures range from unhappy to happy and for the arousal scale, figures range from calm to excited. Participants were instructed to identify themselves corresponding to their place on the continuums. The values for both scales ranged from –4 to 4. In addition, given a number of outcomes to which leads synchronisation, we included a scale for social consequences to investigate the impact of emotional induction and movement in unison on the desire to be engaged with the same individuals in the same activity in the future. The question ranged from 1 to 9 and participants needed to choose a score corresponding to their desire at the moment of being engaged with the same individuals (see Figure 3).

#### Physiological data

We obtained the ECG data with wireless Trigno Avanti Delsys biosensors. Participants placed the electrodes on disinfected skin for better electrical connectivity using the Delsys manual in a modified 3-lead ECG configuration. Electrode V– was placed on the left side below pectoral muscles on the lower edge of the left rib cage. Electrode V+ electrode was placed on the right side below the pectoral muscles on the lower edge of the right rib cage. The sensor body was placed under the positive electrode. R waves are the highest peaks seen on the ECG making them the easiest waves to detect. To be able to analyse heart data, we implemented sym4 wavelet transform to identify the R waves within the QRS complex. Given that individuals vary with regard to their resistance to the current flow as well as slight differences in electrode placements, the obtained ECG signal was of varying amplitude between different people. This caused the peak height to vary between the individuals that do not allow a one-size-fits-all parameter (i.e., the height of the peak and the distance between two successive peaks). To deal with this, as well as with artefacts for pre-processing of the data, we followed a series of steps: firstly, we automatically implemented wavelet transform on all the trials on all the people automatically in a loop. Then, we identified the physiologically implausible values for a moving person with an additional margin (i.e., less than 60 bpm or more than 120 bpm). If the chosen amplitude threshold is too high for a given individual, that will cause not identified peaks leading to a strongly decreased bpm rate. If on the other side the chosen amplitude threshold is too low for a given individual, that would identify by mistake not only the R wave leading to a strongly increased bpm rate. Afterward, these peaks were manually adjusted for each 30-s trial for each individual and compared visually with the raw ECG signal to ensure the correct detection.

Once the R peaks were accurately detected, they were modified into millisecond intervals between two successive heartbeats (i.e., RR intervals). To prepare such a non-stationary signal for phase detection, we transformed the signal. We created a new time series where we allocated number 1 for the time points of identified peaks. Next, we identified the midpoints between each pair of two successive heartbeats and allocated the number –1. This allowed deriving a sinusoidal-like time-series signal prepared for the Hilbert transform implementation. While this transformation brings to the artificial +1 value the heart peaks, it keeps absolutely all information about heart beat intervals and variability identical. It has the advantage that the transformed signal can be processed with continuous signal processing tools (FFT analyses, Hilbert transform method) in order to extract the phase modulation, and allows to interpret results from different levels of analysis (e.g., behavioural, physiological) in the same “language.”

Separate one-way repeated measure ANOVAs were run on each of the metrics of interest, with appropriate *post hoc* comparisons to clarify significant differences between conditions. For this, we averaged different trial values per each emotion nested within each triad (i.e., an average of five trials for each of three emotional conditions). Statistical analyses were performed in the JASP software.

## Results

### Emotion effect on movement synchronisation

**FIGURE 6:**
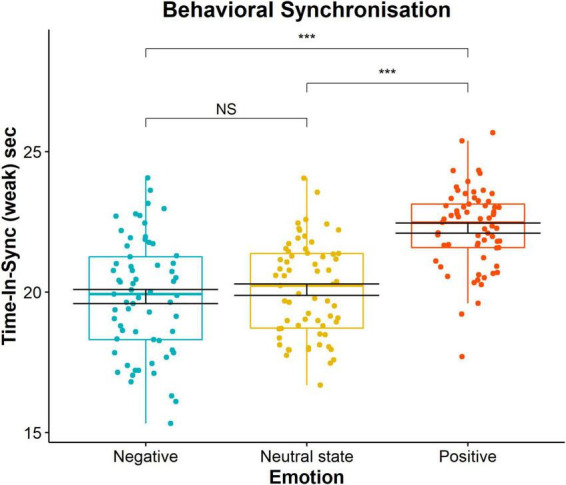
Time in synchrony (s) pairwise comparison for each of the three emotional conditions (negative emotion, neutral state, and positive emotions). Each dot represents a score of one 30 s trial. Significance levels 0.001, 0.01, or 0.05 are indicated with *, **, and *, respectively.

To investigate the impact of emotion, our independent variable, on spontaneous time spent in synchronisation, our dependent variable, we used the TIS metric of the *weak* level. This value was obtained per each trial as a cumulative score for the three participants. The possible range of values is between 0 s (e.g., no attained synchronisation) and 30 s (e.g., synchronisation during the whole duration of the trial). There was a statistically significant main effect of emotion on TIS behavioural scores suggesting that there was a difference between at least two experimental conditions, *F*(2, 24) = 61.65 (*p* < 0.001), η_*p*_^2^ = 0.84. The measures of central tendency and dispersion are as follows: negative emotion (mean = 19.84, SD = 0.41), neutral state (mean = 20.08, SD = 0.67) and positive emotion (mean = 22.28, SD = 0.54). *Post hoc* pairwise *t*-test comparisons with Bonferroni correction revealed that positive emotion was statistically significantly different from negative (*p*_*bonf*_ < 0.001, 95% CI = [–3.06, –1.82]) and neutral state (*p*_*bonf*_ < 0.001, 95% CI = [–2.81, –1.57]) no significant difference between negative emotion and neutral state (*p*_*bonf*_ = 0.952, 95% CI = [–0.87, 3.75]). This indicates that participants spontaneously spent more time in synchrony when they were positively emotionally induced (Figure 6). The effect was non-significant for medium level of TIS *F*(2, 24) = 3.27 (*p* = 0.055), η_*p*_^2^ = 0.21 and remained significant for high level of TIS *F*(2, 24) = 5.87 (*p* = 0.008), η_*p*_^2^ = 0.33.

To test whether this result was due to chance, we carried out pseudo synchrony measures. This approach consists of random reorganisation of individual trials from different triads and computing of the TIS scores. This pseudo behavioural synchrony measure was not significant for any pair of emotions [*F*(2, 24) = 0.14 (*p* = 0.870), η_*p*_^2^ = 0.01], suggesting that the effects of emotion on movement behaviour are not due to chance. With respect to controlling the head orientation, we have arbitrarily chosen a threshold of 0.6 of the values within the dynamic triangle, and we obtained less than 15% of data points larger than the threshold. Given the approximative nature of the threshold, this amount of data is inconsequential and therefore we can conclude that participants complied with the instructions for executing the task and were paying attention to each other.

### Emotion effect on pleasure, arousal, and social consequences

Right after every emotional induction and before the execution of the movement in the next trial, participants reported scores on the Self-Assessment Manikin (SAM) scale. There were two 9-item scales, pleasure and arousal, ranging from –4 to 4. In addition, there was a social consequence on 9 item scale ranging from 1 to 9. The scores for the SAM questionnaire as well as the social consequences item are the dependent variables.

For the SAM pleasure score, there was a statistically significant main effect of emotion on SAM pleasure scores suggesting that there were differences between at least two experimental conditions [*F*(2, 24) = 3.85, *p* = 0.035, η_*p*_^2^ = 0.24]. The measures of central tendency and dispersion are as follows: negative emotion (mean = 1.75, SD = 1.17), neutral state (mean = 2.05, SD = 0.85) and positive emotion (mean = 2.22, SD = 0.86). *Post hoc* pairwise *t*-test comparison revealed significant difference only between negative and positive emotional conditions (*p*_*bonf*_ = 0.034, 95% CI = [–0.92, –0.03]) but not negative and neutral (*p*_*bonf*_ = 0.282, 95% CI = [–0.75, 0.14]) nor neutral and positive (*p*_*bonf*_ = 0.982, 95% CI = [–0.62, 0.27]). The results indicate that positive emotional induction generated perceived higher valence scores and negative emotional induction caused a decline and lowered valence score.

For the SAM arousal scores, the sphericity assumption was violated and therefore the Greenhouse-Geisser correction was implemented. There was no statistically significant difference [*F*(1.38, 16.76) = 2.80, *p* = 0.103, η_*p*_^2^ = 0.19].

For the social consequences scores, there was a statistically significant main effect indicating that there was a difference between at least two experimental conditions [*F*(2, 24) = 13.95, *p* < 0.001, η_*p*_^2^ = 0.54]. The measures of central tendency and dispersion are as follows: negative emotion (mean = 6.28, SD = 1.54), neutral state (mean = 6.64, SD = 1.34) and positive emotion (mean = 6.90, SD = 1.30). *Post hoc* pairwise *t*-test comparisons were significant for negative and neutral (*p*_*bonf*_ = 0.017, 95% CI = [–0.66, –0.05]) and negative and positive (*p*_*bonf*_ < 0.001, 95% CI = [–0.92, –0.32]) but was not different between neutral and positive inductions (*p*_*bonf*_ = 0.105, 95% CI = [–0.56, –0.04]). Negative emotion had the weakest social consequences and positive having the strongest ones.

### Emotion effect on physiology

We transformed the raw inter-beat interval time series into an oscillatory-like time series. This allowed the implementation of Kuramoto analysis and specifically the time series of the order parameter TIS, the dependent variable, per each executed trial as a function of induced emotion, the independent variable. The main effect of emotion on the physiological rhythm entrainment (dependent variable is TIS scores of RR intervals) was also investigated using the one-way repeated measures ANOVA. The results were not significant [*F*(2, 24) = 0.53, *p* = 0.594, η_*p*_^2^ = 0.04]. The effect was neither significant for medium level of TIS [*F*(2, 24) = 1.14, *p* = 0.336, η_*p*_^2^ = 0.09] nor for the high level of TIS [*F*(2, 24) = 0.57, *p* = 0.576, η_*p*_^2^ = 0.05].

## Discussion

The aim of the present study was to investigate the effect of naturalistically induced emotion on synchronisation occurring across behavioural, psychological, and physiological modalities, with positive emotion expected to facilitated the beat in unison between interactants and negative emotion to disrupt it.

### Behavioural modality

We confirmed our hypothesis that individuals induced with positive emotion would spend more time in spontaneous synchronisation than when they were induced with a neutral state and negative emotion conditions. Nevertheless, the negative emotion was not significantly different from the neutral state. These results confirm earlier findings (for instance, Tschacher et al., 2014) and suggest that positive affect is positively correlated to interpersonal movement synchronisation. At the same time, the observed asymmetry between positive and negative inductions contradicts the well-established negativity bias in psychological research (Rozin and Royzman, 2001) that humans are more efficiently induced by negative emotions than by positive ones. It is possible that moving together and just the mere sharing of a physical space facilitate interaction and help people bind together. Presumably, the improvisational type of interaction affords individuals with a drive towards social “likes” to develop strong social ties. There were no significant differences in TIS scores between negative emotion and neutral state. In fact, we have built the neutral condition in such a way that the ambiguity of possibly being the best and the worst at the same time would result in a more neutral induction. It is possible that this would have induced a positive valence in some participants and negative valence in others. However, as differences were found between neutral and non-neutral conditions for some variables (for instance, the social consequence score, see below), this possibility is not entirely convincing.

### Psychological modality

We obtained a significant effect of emotion on pleasure scores but not arousal scores. As mentioned in the introduction, human emotions are not just changes in valence (pleasure scale) but involve a delicate combination between valence, arousal, behaviour and physiology. Given that there were emotional effects not only at the behavioural modality but also at the psychological level, we can conclude that the emotion induction was successful. As for the social consequences, the positive induction increased personal will to engage in a task with the same group members and this was decreased with the negative induction. This is rather a straightforward finding, as it suggests that our social consequences variable can determine the potential benefits of synchrony. What is not yet clear is whether this effect is due to emotional induction, movement synchrony, or both. This is important because it can be used as a tool for developing synergies and strengthening the cohesion levels of a group to attain higher performances.

### Physiological modality

We did not find any significant main effect of emotions on the cardiac activity of the triad. What can be the reason(s) for the absence of an experimental effect on the physiological synchrony, an effect that was found before (e.g., Konvalinka et al., 2011)? One reason may relate to the different metrics used in different studies. For instance, Konvalinka et al. (2011) used a recurrence quantification analysis on cardiac activity, whereas we transformed the signal into a sinusoidal-like signal in order to homogenise our analyses between behavioural and physiological modalities. Another reason relates to the possible impact of physical activity on the Autonomic Nervous System (ANS), which is perhaps larger than the impact of emotion on ANS and could have dominated it. The possible solution is to adapt the task and adopt one that would reduce the movement components to their minimum.

## Conclusion, limitations, and future work

From a theoretical standpoint, this study contributes to verifying whether ecological emotion elicitation can be manifested in human movement and interpersonal motor synchrony and provides a fundamental understanding of how different affective states result in human group synchronisation. From an applied perspective, these results can be used in the rehabilitation context, in which, based on the patient’s movement, synchrony-based recovery interventions can be used to develop rapport (Fuchs and Koch, 2014) and facilitate, for instance, Autism Spectrum Disorder adaptations (Manders et al., 2021). They can also be used to improve human-machine interactions, for example, in adjusting behavioural interactions with human individuals based on their affective state and creating synergies within the group.

One limitation of our study is the possible blending of the effect of two different emotional induction procedures (i.e., deception based on social comparison and affective picture dataset). We cannot completely separate the part of emotion induction that relates to the naturalistic scenario of being evaluated in a personally valuable task by others and the part of emotion induction coming from emotionally inducing pictures. For this reason, a separate study might test each induction method in isolation and then together, preferably within a repeated-measures design to reduce the inter-individual differences. The goal of the present study was not to validate a specific and unique method of induction but rather to attain a high induction efficiency. In this light, the choice of reinforcing manipulated social feedback with an intuitive and fast mean appears justified. In post-experimental debrief, spontaneous feedback provided by a few participants was that they understood immediately from the pictures, without reading the text, whether they were getting a positive or a negative result. In such a manner, it seems that the pictures cued some participants to a specific emotion and helped make the induction instantaneous.

Another limitation of our study is the potential entanglement between emotion and motivation for the chosen experimental task. Indeed, after receiving evaluative social feedback with respect to their own performance, participants could have been more motivated in doing the task, creating a potential confound between emotion and motivation. Against this interpretation are the facts that emotion and motivation are not entirely stackable—the same negative emotion induction for the same individual can arouse anger and increased motivation on one occasion, and can cause sadness and decreased motivation on another occasion—and that a negative induction would have produced an increase in synchronisation, a result that was not found. More, we did tangentially control for the participants’ motivation in our experiment, with the third question asked after each trial “Would you like to continue dancing with the members of this group?” We argue that this question embraces the motivation of individuals to engage in the task by exploring the fluctuations after each emotional induction. Answers to this question were significantly different between emotion conditions (i.e., lowest—negative, middle—neutral state, and highest—positive). Emotion is an emergent phenomenon that builds on motivation, and it is hard to separate them clearly. Emotions are intrinsic in individuals’ achievement patterns (Nicholls, 1984; Dweck, 1986) and are approached as action tendencies (Frijda, 2007), suggesting that each experienced emotion guides and motivates individuals to engage in a specific action (e.g., anger to fight or fear to flight).

Due to the low sample size, care should be taken in generalising the presented findings. In addition, it is possible that emotional induction did not affect all triads and participants in the same way. Nevertheless, our statistical results related to the effect of emotion on behavioural TIS are strong (Figure 6 demonstrates clustering of all data around mean values), indicating that despite interpersonal variability, there was a common and shared tendency toward spontaneous synchronisation.

This research focused on behaviour that combines naturalistic emotion induction and improvisational movements, with a trade-off between ecological validity of emotion induction and experimental control. This highlights the cardinal importance of selecting the best possible trade-off: Ecological validity shall not be sacrificed for the sake of high requirements for experimental control.

In future research, approaching emotion within a repeated-measures design seems a good strategy to be particularly cautious with the emotional aftereffects (Halovic et al., 2020), especially paying attention to the effect of emotions that can build up over time. Notably, emotions are non-linear in nature, meaning that one stimulus does not produce the same reaction between different individuals and even within the same individual on different occasions. Moreover, emotional induction is complicated for the repetitive type of experimental paradigms owing to a gradual individual’s sensitisation to the same stimuli. This formulates a prominent line of research for mutually adapting human-computer and human-robot interaction systems that are occasion sensible and adjustable.

Another avenue when quantifying joint action in general, and synchronisation in particular, is to investigate different emotion entanglement in interpersonal adaptations (Burgoon et al., 1995; Hart et al., 2014). For instance, how much the interaction shapes the individual’s behaviour reflects the degree of behavioural change, which could be informative for illustrating the strength of interaction as a function of emotion. Additionally, the direction of change, or its absence, could reflect leadership processes within group dynamics and whether some emotions overpower others when there are more representatives with one particular emotion rather than another.

## Data availability statement

The datasets presented in this study can be found in online repositories. The names of the repository/repositories and accession number(s) can be found below: https://osf.io/d45t3/.

## Ethics statement

The studies involving human participants were reviewed and approved by IRB 2002A of the EuroMov Digital Health in Motion. The patients/participants provided their written informed consent to participate in this study.

## Author contributions

AS conceived the idea of the manuscript with the help of MB, SJ, and BB, collected the data with the assistance of SP, analysed the data under the supervision of SJ and BB, and wrote the first draft of the manuscript. MB, SJ, and BB contributed to manuscript revision, and read and approved the submitted version. All authors contributed to the article and approved the submitted version.

## Footnotes

1
mentimeter.com
